# Five-year follow-up of secondary iris-claw intraocular lens implantation for the treatment of aphakia: Anterior chamber versus retropupillary implantation

**DOI:** 10.1371/journal.pone.0214140

**Published:** 2019-04-10

**Authors:** Mario Damiano Toro, Antonio Longo, Teresio Avitabile, Katarzyna Nowomiejska, Caterina Gagliano, Sarah Tripodi, Tomasz Choragiewicz, Agnieszka Kaminska, Michele Figus, Chiara Posarelli, Matteo Forlini, Anselm Gerhard Maria Jünemann, Michele Reibaldi, Robert Rejdak

**Affiliations:** 1 Department of Ophthalmology, University of Catania, Catania, Italy; 2 Department of General Ophthalmology, Medical University of Lublin, Lublin, Poland; 3 Institute for Ophthalmic Research, University Eye Hospital, Tuebingen, Germany; 4 Faculty of Medicine, Collegium Medicum, Cardinal Stefan Wyszynski University, Warsaw, Poland; 5 Department of Surgical, Medical, Molecular Pathology and of the Critical Area, University of Pisa, Pisa, Italy; 6 Institute of Ophthalmology, University of Parma, Parma, Italy; 7 Department for Ophthalmology, University Medicine Rostock, Rostock, Germany; Edith Wolfson Medical Center, ISRAEL

## Abstract

**Background:**

Though several procedures of IOL implantation have been described (sutured scleral fixation, intra-scleral fixation, angle-supported anterior chamber, and anterior chamber or retropupillary iris-claw IOLs), there are no randomized trials which are comparing different techniques. Hence, the surgical treatment of aphakia still remains controversial and challenging. The purpose of this study was to compare the long-term efficacy and the rate of complications of anterior versus posterior Iris-claw intraocular lenses (IOL) implantation to correct for the treatment of aphakia without sufficient capsule support.

**Methods and findings:**

Consecutive eyes having secondary implantation of aphakic iris-fixated IOLs with a follow-up of at least 5 years were considered. Mean correct distance visual acuity (CDVA) changes, percentage of eyes with CDVA improvement, mean corneal endothelial cell density (CECD) loss and the rate of other complications were used for statistical analysis. The study evaluated a total of 180 eyes (Group A: 87 anterior chamber iris-claw fixation, Group B: 93 retropupillary iris-claw implantation) of 180 consecutive different patients, with aphakia of various reasons. CDVA improved significantly in both groups after surgery (P<0.001, ANOVA), and was remarkably higher than baseline in both groups from first week and during the entire follow-up (P<0.001, Tukey’s Honest Significant Difference). There was no statistically significant difference in CDVA between the two groups during each follow-up visits (P = NS, unpaired t-test) and in the CDVA improvement percentage between the two groups (P = 0.882, Chi-square test). No significant changes in CECD were noted after surgery in both groups (ANOVA Group A: P = 0.067, Group B: P = 0.330P). No intra-operative complications occurred in both groups. There was no statistically significant difference in the rate of complications between the two groups (P = NS, Chi-square test), except for pigment precipitates which were higher in Group A (P<0.05, Chi-square test).

**Conclusions:**

Five-year follow-up shows that secondary implantation of aphakic IOLs is effective and safe for the correction treatment of aphakia in eyes without capsule support.

## Introduction

Aphakia, with an inadequate capsular support for in-the-bag or sulcus intraocular lens (IOL) implantation in the bag or ciliary sulcus, may be the result of complicated cataract surgery, lens dislocation or trauma [1]. Nowadays, the surgical treatment is controversial and remains a challenge. Though several procedures of IOL implantation have been described (sutured scleral fixation, intra-scleral fixation, angle-supported anterior chamber, and anterior chamber or retropupillary iris-claw IOLs) [1–3], there are no randomized trials which are comparing different techniques. Hence, the optimal choice for an IOL implant is frequently focused on the eye’s status and surgeon’s experience [4].

Several complications have been reported about each of these secondary implants in previous studies. The most common complication of angle-supported anterior chamber IOLs was bullous keratopathy, followed by lens dislocation, secondary glaucoma, macular edema and retinal detachment [5]. Scleral-sutured IOLs offer the advantage of fixation in the posterior chamber, but the surgical technique is technically more difficult and requires a longer surgical time. Inadequate fixation of the scleral sutures can be associated with lens tilt, suprachoroidal and vitreous hemorrhage, or retinal detachment. Moreover, erosion of conjunctiva with exposition of the fixation suture may be associated with an increased risk of endophthalmitis, and the breaking of the suture can lead to IOL dislocation [6].

For these reasons, iris-claw aphakic IOLs are actually considered as the best choice for secondary implantation in adult patients by many surgeons. In the early 1960s, Collar implanted the first iris-fixated lens after an intra-capsular cataract extraction, but in 1971, Worst came in with the Iris Claw lens, and its modification evolved in the Artisan lens [4,7]. Actually iris-claw IOL implantation is considered as an effective, predictable and safe option for aphakic eyes without capsule support, with a quicker visual recovery, better better visual outcomes and fewer complications than the other secondary implants. Furthermore, its placement can be performed with a lower invasiveness and in a shorter surgical time than the others [8–10]. Iris claw IOLs have been successfully implanted either in the anterior or in the posterior chamber [2]. However there is no general consensus about the best placement.

To date, only one prospective study have compared the anterior and posterior secondary iris claw fixation, but this trial involved a relatively small-size sample for a short follow-up; and also no data have been reported on the corneal endothelial cell density (CECD) and the long-term incidence of complications. So far, that study has not resolved safety-related problems [10].

The aim of our study was to compare the long-term efficacy and the rate of complications of anterior and posterior Iris IOL implantation for the treatment of aphakia without sufficient capsule support.

## Methods

### Patients and methods

In this retrospective study, we have included all consecutive aphakic eyes without capsular support and who received Artisan iris-claw IOL (Ophtec BV, Groningen, The Netherlands) as secondary IOL implantation at the Department of Ophthalmology, University of Catania (Italy) from February 5, 2008, to February 5, 2013.

The study protocol, was approved by the Local Ethics Committee of the Azienda Policlinico Vittorio Emanuele Rodolico, Catania, Italy, confirmed the tenets of the Declaration of Helsinki. A written informed consent form for the processing of personal data was obtained from all patients.

#### Inclusion criteria

Aphakia which was due to trauma, complicated cataract surgery and lens/IOL luxation, integrity of the iris which allows to enclavation of the IOL's claw, anterior chamber depth >3.2 mm, endothelial cell density >900/mm^2^, intraocular pressure (IOP) ≤ 21 mmHg, normal retinal examination and a follow-up of at least 5 years after the surgical procedure.

#### Exclusion criteria

Iris defects, glaucoma, uveitis, retinopaties and any ocular co-morbidity that was judged to interfere with the improvement in visual acuity.

If both eyes of the same patient had undergone iris-claw IOL implantation, only 1 was randomly selected for inclusion.

All enrolled eyes were divided into 2 groups: anterior chamber (Group A) and retropupillary (Group B) implantation group. Group A received Artisan iris-claw IOL (Ophtec BV, Groningen, The Netherlands) implantation over the iris, and Group B also received same Artisan iris-claw IOL in a face down retropupillary implantation.

Patient demographics (such as age, gender, and etiology of aphakia) and ophthalmic preoperative and postoperative data were abstracted from the electronic medical records. Two separate abstractors (who had been trained in the methods of chart abstraction) reviewed the charts of patients independently. Definitions for key variables and all data abstraction forms were reviewed. Chart abstractors were masked to the study hypothesis.

Corrected distance visual acuity (CDVA), slit-lamp biomicroscopy examination, gonioscopy, IOP, central corneal thickness (CCT), corneal endothelial cell density (CECD), central macular thickness (CMT) and fundus examination were evaluated preoperatively and after 1^st^ week, 1^st^, 3^rd^ and 6^th^ month, 1^st^, 3^rd^, and 5^th^ year postoperatively.

The CDVA was measured using the Snellen chart and was converted to a logarithm of the minimum angle of resolution (logMAR) visual acuity for calculation. Each line on the chart represents a change of 0.1 log unit in the acuity level with a value of 0.02 log unit for each letter. A change of at least 0.1 log units (≥ 5 letters) was considered statistically significant. A CDVA of 20/2000 and 20/20000 were considered equivalent to counting fingers and hand motion at 2 feet, respectively.

IOP was measured using a Goldmann applanation tonometry. After surgery, a value of IOP >21 mmHg was considered eligible for topical treatment with hypotensive drugs. Preoperative anterior chamber depth and intraocular IOL position at 1^st^ month after the surgery were assessed with ultrasound bio-microscopy (UBM, ParadigmMedicalIndustries, Salt Lake City, Utah). The CCT was assessed with Orbscan II (Bausch & Lomb, Inc., Rochester, New York, USA). All examinations were performed by the experienced operators based on a manufacturer-recommended acquisition protocol. Proper care was taken to obtain a good centred and aligned scan. For each eye, at least three valid assessments with wide corneal coverage were obtained: the measurement with the least eyelid shadow was chosen for the analysis. Patients wearing contact lenses were advised to stop their use respectively 2 weeks or 4 weeks before the IOL implantation. The CECD were measured by Corneal Confocal Microscope (Confoscan 4.0, Nidek Technologies, Italy) during each visit. By semi-automated technique, automatic cell outlines were reviewed and then corrected manually. In each eye, measurement of CECD was made in two endothelium images and the mean value was used for data analysis. The CMT was measured by Stratus OCT (OCT3; Zeiss-Humphrey, San Leandro, CA, USA). All the intra-operative and postoperative complications were noted.

#### Surgical technique

The Artisan aphakia IOL (Ophtec BV, Groningen, The Netherlands) having polymethyl methacrylate IOL with 8.5-mm length, 1.04 mm maximum height, and 5.4-mm optical zone width was used as the lens. The optic power was calculated using the SRK/T formula with the aim of achieving emmetropia. IOL power as per manufacturer’s recommendation was calculated with an A-constant of 115.0 for Group A and 116.5 for Group B, by ultrasonic biometry (QUANTEL CINESCAN S -A/B SCAN, Cournon d'Auvergne Cedex, France). All surgeries were performed by the same surgeon under the peribulbar or subtenon anesthesia.

Two side-ports were made at 3 and 9 o’clock positions. Anterior vitrectomy was performed wherever it was required. Miosis was achieved by injecting intra-cameral acetylcholine 1%, subsequently cohesive viscoelastic was injected. Superior limbal corneal incision of 5.5 mm was designed and the Artisan IOL with the vault facing up for the anterior clawed lens, and vault facing down for the retropupillary lens was introduced into the anterior chamber. The IOL was rotated such that the haptics were positioned at 3 and 9 o’clock. Thereafter, Artisan IOL's optic was held with its special Artisan lens forceps; for anterior chamber group, iris was enclavated at mid-periphery between claw haptics using special enclavation micro spatula. In contrast, for the retropupillary fixation, after repositioning of one haptic, the IOL behind the iris was enclavated using the micro spatula, followed by enclavation of the other haptic. Wherever pupil was minimally distorted (i.e. ovalisation of the pupil), light diathermy application was performed to the anterior iris tissue for contraction of tissue. This technique was aimed at achieving immediate post-operative circular pupil. Superior peripheral iridectomy was performed for only those with anterior iris claw IOL implantation. At the end of this procedure the corneal incision is apposed with interrupted 10–0 non-absorbable nylon sutures. Viscoelastic material was aspirated, and sub-conjunctival gentamicin and dexamethasone were injected. Sutures were removed approximately after 2 months. All the patients were prescribed to prednisolone acetate in tapering fashion and moxifloxacin drop 5 times/day for a week.

#### Statistical analysis

The values of CDVA, IOP, CCT, CECD and CMT were expressed as mean ± standard deviation (SD). In each group, CDVA, IOP, CCT, CECD and CMT mean values were determined before and after IOL implant by ANOVA based comparison; if significant, the difference vs. baseline value was tested by Tukey HSD (Honest Significant Differences) test. Unpaired t-test was used to compare the values of the various parameters detected in both the groups at each time point. Comparison of indications and complications were analyzed with Chi-square test. Statistical significance was set at P<0.05. All the data were analyzed using the Statistical Package for the Social Sciences (SPSS), v.17.0 for Windows (SPSS, Chicago, Ill., USA).

## Results

Overall 180 aphakic eyes (Group A: 87 anterior chamber iris-claw fixation, Group B: 93 retropupillary iris-claw implantation) of 180 patients (102 males, 78 females) met the inclusion criteria and were enrolled in the study. Mean age of the patients was 70 ± 6 years (range: 51–85 years). The demographics and etiology of aphakia in both groups are summarized in Table 1.

**Table 1:** Demographics and etiology of aphakia for Group A (anterior chamber iris-claw fixation) and Group B (retropupillary iris-claw implantation).

Characteristics	Group A	Group B	P
	(n = 87)	(n = 93)	
Gender, n (%)			
Male	49 (56%)	53 (57%)	1.000*
Female	38 (44%)	40 (43%)	
Eye, n (%)			
Right	47 (54%)	41 (44%)	0.185*
Left	40 (46%)	52 (56%)	
Age (Mean ± SD) (years)	70.6 ± 5.5	69.5 ± 6.3	0.215°
Etiology, n (%)			
Spontaneous or traumatic lens/lOL subluxation	6 (7%)	6 (6%)	0.862*
Complicated cataract surgery resulted in aphakia	42 (48%)	47 (51%)	0.888*
Peroperative lens/IOL luxation	33 (38%)	35 (38%)	0.920*
Previous intracapsular cataract extraction	6 (7%)	5 (5%)	0.920*

SD = standard deviation.

*Chi-square test

°t-test

The most common etiologic factor was complicated cataract surgery (48% for Group A and 51% for Group B).There was no significant difference in indications in two groups (P = NS, Chi-square test).

### CDVA

CDVA improved significantly in both groups (P<0.001, ANOVA), from first week and during the entire follow-up (P<0.001, Tukey HSD). There was no significant difference observed in the mean CDVA between the two groups at each follow-up visit (Table 2). At the last visit, in Group A, 47 eyes (54%) achieved better CDVA values, 34 eyes (39%) had the same CDVA and 6 eyes (7%) had a poorer CDVA in comparison to preoperative data. In Group B, at the final follow-up, 49 eyes (52.5%) achieved a better final CDVA values, 37 eyes (40%) had the same CDVA and 7 (7.5%) patients had a poorer CDVA in comparison to preoperative data. There was no significant difference observed in the rate of eyes with CDVA improvement between two groups (P = 0.882, Chi-square test).

**Table 2:** Mean CDVA ± SD (logMAR) of Group A (anterior chamber iris-claw fixation) and Group B (retropupillary iris-claw implantation).

Category	Pre-operative	Post-operative						
		1st week	1st month	3rd month	6th month	1st year	3rd year	5th year
Group A (n = 87)	0.37±0.21	0.15±0.21*	0.13±0.19*	0.14±0.23*	0.13±0.22*	0.12±0.16*	0.12±0.11*	0.12±0.15*
Group B (n = 93)	0.41±0.22	0.17±0.23*	0.15±0.21*	0.13±0.15*	0.12±0.14*	0.13±0.13*	0.14±0.13*	0.13±0.15*
P (Unpaired t-test)	0.214	0.544	0.504	0.786	0.712	0.645	0.268	0.655

CDVA = correct distance visual acuity

SD = standard deviation

logMAR = the minimum angle of resolution

ANOVA: both groups P<0.001

*Tukey HSD vs. baseline all measurements: P<0.001

### IOP

IOP increased significantly among both the groups at 1^st^ week and 1^st^ month (both ANOVA P<0.001, Tukey HSD P<0.001). A IOP>21 mmHg was noted in 16 patients in Group A and in 21 patients in Group B at 1^st^ week postoperatively. Patients received a mono-therapy (dorzolamide or timolol) if 21 < IOP ≤ 25 mmHg, or a dorzolamide/timolol fixed combination administered twice daily if IOP>25 mmHg; no one had a permanent increase of IOP. There was no significant difference observed in IOP between the two groups at each follow-up visit (Table 3).

**Table 3:** Comparison of mean IOP ± SD (mmHg) of Group A (anterior chamber iris-claw fixation) and Group B (retropupillary iris-claw implantation).

Category	Pre-operative	Post-operative						
		1st week	1st month	3rd month	6th month	1st year	3rd year	5th year
Group A (n = 87)	14.4±2.35	18.7±1.57*	16.1±1.49*	14.9±1.33	14.8±1.22	14.6±1.19	14.7±1.20	14.6±1.18
Group B (n = 93)	14.9±1.42	19.1±1.42*	16.5±1.62*	15.2±1.55	15.1±1.42	14.6±1.30	14.8±1.23	14.7±1.31
P (Unpaired t-test)	0.084	0.074	0.087	0.166	0.131	1	0.582	0.592

IOP = intraocular pressure; SD = standard deviation; mmHg = millimeter of mercury

ANOVA: both group P<0.001

Tukey HSD vs. baseline *P<0.001

### CECD

CECD did not change in both the groups after surgery (ANOVA, Group A; P = 0.067, Group B; P = 0.330). The mean difference in CECD before and at 1^st^ week, 1^st^ month and 5^th^ year after surgery was 92 (4,.9%), 107 (5,.7%) and 219 (11,.6%) cell/mm^2^ in Group A respectively, and 110 (5.9%), 135 (7.3%) and 176 (9.5%) cell/mm^2^ in Group B, respectively. No significant difference in CEDC was observed between the two groups at each follow-up visit (Table 4).

**Table 4:** Comparison of mean CECD ± SD (cell/mm^2^) and mean difference in CECD (%) of Group A (anterior chamber iris-claw fixation) and Group B (retropupillary iris-claw implantation).

Category	Pre-operative	Post-operative						
		1st week	1st month	3rd month	6th month	1st year	3rd year	5th year
Group A	1872±460	1780±479	1765±463	1735±490	1699±497	1677±487	1684±502	1653±477
		-92(4.9)	-107(5.7)	-137(7.3)	-173(9.2)	-195(10.4)	-188(10)	-219(11.6)
Group B	1845±521	1735±462	1710±501	1707±515	1700±508	1694±476	1679±503	1669±493
		-110(5.9)	-135(7.3)	-138(7.5)	-145(7.8)	-151(8.2)	-166(9)	-176(9.5)
P (Unpaired t-test)	0.714	0.522	0.446	0.71	0.989	0.813	0.947	0.526

CECD = corneal endothelial cell density; SD = standard deviation; cell/mm2 = number of cells per mm^**2**^

### CCT and CMT

CCT and CMT did not change after surgery in both groups (ANOVA, NS) and there was no significant difference between two groups at each follow-up visit (paired t-test, NS).

### Complications

No intra-operative complications occurred in any of our cases. Postoperative complications are shown in detail in Table 5. Pigment precipitates were found mainly in Group A (P = 0.009, Chi-square test); the other complications had the same rates in both groups (Chi-square test, P>0.05). No cases of corneal de-compensation were noted during the follow-up. All the IOLs were well-centered 1 week postoperatively. Nevertheless, pupillary ovalization was seen in 1 patient in Group A and in 2 patients in Group B; these distortions were ended within a week. One eye of Group B developed a retinal detachment 3 years after surgery and underwent 25-gauge vitrectomy with gas tamponade. The patient referred a head-trauma occurred five days before the visit.

**Table 5:** Postoperative complications for Group A (anterior chamber iris-claw fixation) and Group B (retropupillary iris-claw implantation).

Complication	Group A	Group B	P*	Comments/Management (n° of Eyes)
Early (1st week-3rd month)				
Wound leak	3 (3,4)	2 (2,1)	0.92	Conservative treatment (3); resuturing (2, one for group)
Hyphema	3 (3,4)	2 (2,1)	0.92	Observation
Pupil ovalization	1 (1,1)	2 (2,1)	1	Observation
IOP elevation	16 (18,4)	21 (22,5)	0.61	Topical medication (37)
Pigment precipitates	26 (29,9)	12 (12,9)	0.009	Topical medication (38)
Hypotony	1 (1,1)	1 (1)	0.507	Wound leak resuturing (2)
CME	1 (1,1)	2 (2,1)	1	Topical medication (3)
Late (1st-5th year)				
Elevated IOP	1 (1,1)	1 (1)	0.507	Topical treatment (2)
IOL dislocation	1 (1,1)	2 (2,1)	1	At 1 yr (2, one for group), at 3 yr (1, group B); all repositioned
Pigment precipitates	5 (5,7)	3 (3,2)	0.647	Observation
Retinal tear	0 (0)	1 (1)	1	At 2 yr; laser treatment
Retinal detachment	0 (0)	1 (1)	1	At 3 yr; 25-gauge vitrectomy with gas tamponade

* Chi-square test

## Discussion

Our study indicates that both the anterior and posterior Iris IOL implantation is effective in the treatment of aphakia without sufficient capsule support by improving visual acuity significantly without serious intra-operative and postoperative complications at the five-year follow-up.

There is still no established consensus on the best choice of treatment to correct aphakia without a sufficient capsule support [1,11]. Nowadays, the major possibilities for secondary IOL implantation are: transclerally sutured posterior chamber IOLs (PC IOLs) [8], angle-supported anterior chamber IOLs (AC IOLs) [12], iris-fixated IOLs introduced through the scleral tunnel [7,13–14] and the flanged IOL fixation technique by Yamane et al. [15]. However, iris-fixated IOLs are usually preferred by surgeons because they yield an early visual recovery and better visual outcomes that can be performed less invasively and in a shorter surgical time [9]. They also have a lower incidence of intra-operative and post-operative complications than the other two IOL types [7, 16–18]. However, there is no evidence available about the best placement method for secondary iris-claw IOL implantation that offers the maximum and earliest visual recovery over many years and the lowest complication rate [6,19]. Despite a higher incidence of IOL dislocation [11], the retropupillary fixation offers the advantage with physiological posterior chamber implantation, resulting in a deeper anterior chamber and a lower intra-operative and postoperative risk of corneal de-compensation than anterior fixation [11,20,21]. However the published literature on iris-claw IOLs in aphakia is limited with relatively small numbers of patients with a short follow-up [1,8,17,22–24].

In this retrospective study, we have compared the long-term efficacy and the rate of complications for both anterior and posterior Iris IOL implantation to treat aphakia without sufficient capsule support.

Regarding efficacy, in Group A and B the mean CDVA improved significantly from 0.37 ± 0.21 and 0.41 ± 0.22 logMAR preoperatively to 0.15 ± 0.21 and 0.17 ± 0.23 logMAR 1 week after surgery, respectively. After the first month, there was no difference in the postoperative CDVA. In addition, the postoperative CDVA was equal to or better than the preoperative CDVA in 93% and 92.5% of eyes in Group A and B respectively. Our results are similar to those of other studies of iris-claw IOLs [1,7,4,9,11,22] and better than those obtained with iris-sutured PC IOLs, secondary open-loop AC or sulcus-sutured PC IOLs [25–30].

The limitation of iris claw IOL, either anterior iris or posterior iris position, includes the 5.5 large incision and the consequent corneal astigmatism. Baykara et al. preferred a scleral tunnel incision with a surgical procedure that normally does not require sutures [21]. Peralba et al. in their retrospective comparative study on iris-claw IOLs reported that creating a wide clear corneal incision could cause a high level of astigmatism if not adequately managed [31].

All the patients included in our study were operated by the same anterior segment surgeon who performed a superior limbal corneal incision of 5.5 mm. Superior incision is better protected by the superior eyelid while large temporal incision is more exposed to trauma and infections. Precautions were taken to obtain a residual astigmatism after suture removal which was better tolerated. The surgeon who was accustomed to manage the corneal astigmatism was preferred to suture the limbal corneal wound with three 10–0 nylon sutures and selectively removed them after 2 months, depending on the patient’s refractive and topographical astigmatism.

However, de Silva et al. have confirmed that it is difficult to assess the functional outcomes for each IOL type, reported in the literature, because these lens are often implanted in eyes with ocular co-morbidities which may themselves limit the final visual outcomes or after complicated cataract surgery, which may itself cause complications [1].

One primary concern about aphakic iris-fixated IOL implantation is the loss of corneal endothelial cells [7,32,33] and it has been reported to be similar to that of routine cataract operation [34]. In our study, the mean CECD decreased by 9 to 10% three years after surgery which is comparable to the results obtained by other studies [8,35]. Anbari and Lake reported a postoperative decreasing of manual endothelial cell density of about 12% at two-years after retropupillary iris-claw implantation [36]. According to Gicquel et al., the anterior chamber implantation causes a higher endothelial cell loss than the retropupillary one [20]. In our series we had only two cases with an endothelial cell density of <1000 cell/mm^2^ which received a retropupillary fixation in both cases. However we preferred to implant IOL retropupillary when the ECC was less than 1200 cells/mm^2^. This surgical approach has been also followed by Peralba et al. in a recent retrospective case series where authors compared outcomes related to the two surgical techniques of implantation [31].

The mean difference in CECD before and at 1^st^ week and 1^st^ month after surgery was 92 (4,9%) and 107 (5,7%) cell/mm^2^ in Group A, and 110 (5.9%) and 135 (7.3%) cell/mm^2^ in group B, respectively, suggesting that most endothelial cell damage occurs intra-operatively. However Qasem et al. reported that endothelial cell loss rate is meaningless at five years follow-up [37], with an increase of 1.6% and 0.5% for Group A and Group B respectively from 3^rd^ to 5^th^ year. Moreover, in our series there was no evidence of corneal de-compensation during the entire follow-up in both the groups. Yueqin C et al. [7] suggested that the corneal endothelial cell loss may be mainly because of a mechanical injury due to the contact which occurs during the surgical procedure between the endothelium and the instruments or the IOL. This risk can be avoided or limited by protecting the endothelial cells with an adequate amount of viscoelastic material during the surgery. In aphakic iris fixated IOLs, Jonker et al. [38] identified two main risk factors for endothelial cell loss: a shallow anterior chamber depth and a smaller distance between the central and peripheral IOL edge to the endothelium. Baykara et al. [21] suggest that implanting an Artisan aphakic IOL through a scleral tunnel incision results in a less endothelial damage than through a clear corneal incision. To reduce this risk, we performed a limbal corneal incision in all cases and iris-claw IOLs were implanted only in case of aphakic eyes with a deep anterior chamber (>3.2 mm).

As reported previously by Helvaci S. et al. [10], all eyes achieved the desired anatomic results and no intra-operative complications occurred in any of our cases.

The main postoperative complications reported with Artisan iris-fixated IOLs include: IOP elevation, glaucoma, pupillary block, pupil ovalization, wound leak, IOL dislocation, hyphema, retinal detachment, and cystoid macular edema [7,8,35].

Elevated IOP were noted in 16 patients in Group A and in 21 patients in Group B 1^st^ week post-operatively, without significant difference in IOP means between the two groups during each follow-up visit. All cases had topical hypotonizing therapy and no prolonged IOP increase was observed. These results were consistent with the data of Helvaci et al. [10].

Two eyes, one in each group developed a late IOP elevation at 3^rd^ year follow-up visit and after which a topical therapy was started. Both of these patients had a family history for primary open angle glaucoma without any inflammation in the anterior chamber or irido-corneal angle synechiae.

In agreement with de Silva et al. [1], the incidence of lately increased IOP in both groups was comparable to that which was found after a secondary AC IOL fixation (from 0% to 7%) [26,39] and lower than the value after a secondary sulcus-sutured (from 0% to 30.7%) [17,27] or iris-sutured PC IOL fixation (from 5% to 30%) [29]. The complete anterior vitrectomy performed probably prevented a permanent IOP increase in our series. We performed the peripheral iridectomies only in Group A and no cases of pupillary block occurred. As suggested by Forlini et al. this could be explained by the posterior vaulting design of this IOL, when fixed in a reverse position retropupillary, and the space between the back of the iris and the IOL optic [2].

A day after surgery in our series, a total of five eyes (3 from Group A and 2 from Group B) had a wound leak which required a resuturing in two cases (one for each group) for the presence of hypotony and a shallow chamber. As suggested by de Silva et al. [1], a higher incidence of wound leak is expected in these cases as a consequence of a 5.5 mm corneal incision in eyes with previously incised cornea. To ensure sufficient corneal healing, we roughly left corneal sutures *in situ* for 2 months before their removal.

Persistent ovalisation of the pupil has been reported from 0% to 13.9% of eyes, especially in patients who underwent iris reconstruction and more frequently after posterior iris-claw IOL implantation [2,10,11,21]. In our study, this complication was temporarily (<1 week) observed in three patients (1 in Group A and 2 in Group B). To avoid this complication, wherever pupil was minimally distorted, the light diathermy application was performed on the anterior iris tissue for contraction of the tissue.

Another challenge of retropupillary implantation is the probability of IOL dislocation into the vitreous cavity when enclavation fails, especially after severe trauma [40]. However, these cases can be often treated by re-enclavation [1]. de Silva et al. reported 6% total incidence of iris-claw IOL dislocation, occurring from 5 days to 60 months after insertion and often due to the rigid claws in the earlier generation IOL models [1].

In our series we observed a dislocation rate of 1.6% i.e., 2 cases one for each group of spontaneous dislocation at 1^st^ year of follow-up and 1 case occurring after a head-trauma 3 years after the surgery in Group B. All cases were successfully repositioned. Our rate was comparable to the one reported by other studies of posterior-fixated iris-claw IOLs (0% to 10%) [41–43], and lower than the dislocation rate due to suture breakage in scleral-fixated PC IOL (between 7.8% and 27.9%) [12,44]. It has been suggested that incidence of iris-claw IOL dislocation is higher in IOLs inserted earlier in the series, suggesting a learning curve [1]. Our lower rate could be due to well experienced/trained surgeon.

A retinal detachment percentage of 6.3% to 8.2% and a choroidal hemorrhage percentage of 3.2% after the implantation of a trans-sclerally sutured PC IOL were reported by Vote et al. [44] and Bading et al. [12]. In agreement with other studies [1,10,23,41,45], we have not observed any such rate of these complications after both techniques. In our series only a traumatic retinal detachment occurred in a patient who had received retropupillary implantation 3 years after the surgery.

Another drawback is the possibility of Artisan IOL damage on the iris [21]. In our study, to reduce the incidence of pigment precipitates at the end of IOL implantation, we had administrated a sub-conjunctival corticosteroid [46]. Notwithstanding, we observed various degrees of pigment precipitates on the surface of the IOL in all eyes 1 day after surgery, that disappeared after topical corticosteroid treatment. During the last follow-up there was a statistically significant difference observed between the two groups, for pigment precipitates (P<0.05, Chi-square test), which were present in 5 eyes in Group A and in 3 eyes in Group B. As shown previously [7] in our study no cases of pigment erosion, progressive pigment dispersion or iritis have been observed.

In our study the total incidence of CME was 1.6% at third month, with a chronic CME incidence of 0%, which is comparable if not better than the rates reported in the literature for each technique. These rates range from 0% to 33% in case of secondary open-loop AC IOL [26,47], with higher rates after complicated cataract surgery, from 0% to 7.6% after sulcus-sutured PC IOLs [17,27], from 0% to 16.7% after iris-sutured PC IOLs [30], and from 5.8% to 33% after implantation of scleral-fixated PC IOLs [48,49]. Our rate is even lower than the rate (from 4.1% to 4.8%) reported in other studies after retropupillary iris-claw implantation [41,43].

## Conclusions

The study suggests that both anterior and retropupillary implantation of Artisan IOL are effective in visual improvement with a lower rate of complications. In consideration of these results, both anterior and retropupillary implantation of Artisan IOL are easily applicable surgical procedures. The choice of surgical procedure depends on the experience of the surgeon. Moreover, further prospective and randomized studies are required to compare the anterior and posterior implantation of Artisan IOL with a larger sample group and long-term follow-up with the patients.
